# Performance of fissure sealants on fully erupted permanent molars with incipient carious lesions: A glass-ionomer-based versus a resin-based sealant

**DOI:** 10.34172/joddd.2020.009

**Published:** 2020

**Authors:** Nada Jaafar, Hala Ragab, Ahmed Abedrahman, Essam Osman

**Affiliations:** ^1^Department of Pediatric Dentistry, Faculty of Dentistry, Beirut Arab University, Lebanon; ^2^Department of Operative and Esthetic Dentistry, Faculty of Dentistry, Beirut Arab University, Lebanon; ^3^Department of Pediatric Dentistry and Dental Public Health, Department of Pediatric Dentistry and Dental Public Health, Faculty of Dentistry, Alexandria University, Egypt; ^4^Department of Dental Materials, Faculty of Dentistry, Beirut Arab University, Lebanon

**Keywords:** Fissure sealant, glass-ionomer, non-cavitated occlusal caries, sealants

## Abstract

***Background.*** The effectiveness of fissure sealants in caries prevention depends on their long-term retention and ability to stop caries progression. This randomized controlled clinical trial compared the retention rate and cariostatic properties of a contemporary glass-ionomer-based sealant (GIS) versus a resin-based sealant (RS) placed on fully erupted permanent molars in a split-mouth design.

***Methods.*** The sealants were placed on fully erupted permanent teeth (8‒12 years of age) in 45 children. The evaluation was conducted after one week and three and six months.

***Results.*** There was a statistically significant difference in the retention rate and caries transition between the two groups over a six-month clinical evaluation period. The resin-based sealant group showed a better retention rate than the GIS group (75.56% and 48.88%, respectively). The resin-based sealant was superior to GIS in preventing caries progression.

***Conclusion.*** Resin-based fissure sealant with fluoride releasing properties might be preferable in preventing caries progression of incipient non-cavitated carious lesions in fully-erupted teeth.

## Introduction

The occlusal surface of molars is accountable for 67‒90% of dental caries in school-aged children from 5 through 17 years old.^1,2^ The complex morphology of the occlusal pits and fissures warrants an ideal site for the retention of bacteria and food remnants, rendering proper oral hygiene maintenance difficult. Another factor that is responsible for the high incidence of occlusal caries is the lack of salivary access into the fissures due to surface tension, preventing remineralization and thus lessening fluoride effectiveness at this spot as compared with the smooth surfaces.^3^ Therefore, researchers have tried to develop efficient and effective treatments to prevent high-risk children from developing caries, especially soon after their first teeth erupt.

Sealants have been shown to protect the occlusal surfaces, inhibit bacterial growth, and provide a smooth surface, thus increasing the probability that the surface will stay clean. Researches have clearly demonstrated that sealants can be used therapeutically over non-cavitated carious lesions based on the fact that caries is driven by the biofilm on the surface of the lesion; if all the dental plaque is removed or the carious lesion is isolated from the biofilm, then caries will arrest. Therefore, when dealing with occlusal caries, the clinician should follow the ‘if-in-doubt-seal’ management strategy, as the evidence indicated that this would be effective and in the best interest of the patient.^4^

Over the past 30 years, various materials and techniques have been developed to improve pit-and-fissure sealant quality and longevity. Traditionally, RS has been placed as the most commonly used sealant material. The effect of this material relies on its micro-retention due to the creation of enamel tags after acid etching. However, RS is moisture-sensitive, and under wet conditions, especially in children, GIS might be more useful due to its hydrophilic characteristics. The caries preventive and arresting effect of GISs has been credited to its adhesion due to calcium bonds and its ability to leach fluoride into the oral cavity.^5^ Numerous clinical studies have confirmed the effectiveness of both RS and GIS in caries prevention. Although the retention rate of RS is higher than that of GIS, the caries-preventive effects of both materials are similar. This might be due to the fact that the caries-preventive effects of GIS are related not only to the retention of the material but also to its biologic properties. It is worth mentioning that most studies were conducted on partially erupted posterior teeth were isolation might be more complicated. The material behavior might be different when teeth are fully erupted and in occlusion. This clinical aimed to assess and compare sealant retention and caries transition of a GIS versus an RS placed on fully erupted non-cavitated occlusal carious lesions in permanent teeth using a split-mouth design over six months. The null hypothesis was that there would be no difference in the clinical performance of the two fissure sealants.

## Methods

This clinical trial, with a comparative design, was carried out in the specialty clinics, Faculty of Dentistry, Beirut Arab University, Lebanon, after the approval of the Ethics and Research Committee and Beirut Arab University Institutional Review Board (code: 2018H-0058-D-P-0258). The number of children was determined according to the sample size calculation website: htpp://epitools.ausvet.com.au (Ausvet), by considering the means (2.2‒1.5) and pooled variance (1.44) from a previous study conducted by Prathibha et al^27^ (2019) on retention of resin and glass-ionomer sealants on permanent teeth. Assuming a confidence level of 95% and a study power of 80%, the calculated sample size was 74 teeth. It was increased by 20% to eliminate the probability of dropout through the treatment period. Thus, a total of 90 fully-erupted early permanent teeth were recruited conveniently from 45 children, fulfilling the inclusion and exclusion criteria. The randomization process was performed by a toss of a coin, and the unit of randomization was the side of the mouth. Patients selected from the outpatient clinic were 8‒12 years of age. All the selected individuals were healthy, had bilateral fully erupted molars or premolars with non-cavitated incipient carious lesions on the occlusal surfaces (an ICDAS code of 1‒4) (Table 1).^6-8^ The selected teeth were free from restorations, hypoplasia, fractures, or cracks.^9^

**Table 1:** The International Caries Detection and Assessment System (ICDAS) scoring system

**ICDAS score**	
**1**	**Visual change seen in the enamel of pit or fissure areas after air drying.**
**2**	**Distinct visual changes seen in enamel when wet: white or colored, wider than the fissure/fossa.**
**3**	**Localized enamel breakdown without visible signs of dentinal involvement**
**4**	**Underlying dark shadow from dentine**

Uncooperative patients, patients with special needs, or patients having received professional fluoride application within the last six months were excluded from this study. The objectives, risks, and benefits of the study were explained to the parents/guardians, and a signed informed consent form was obtained prior to treatment.

### Clinical procedures

The teeth were visually inspected after proper drying under a standardized light source using the International Caries Detection and Assessment System (ICADs). A WHO probe was passed on all the pit and fissure surfaces, starting from the mesial to the distal side of the occlusal surface.^4^ The sample was divided randomly into the study site and control site, each consisting of 45 teeth. All the clinical procedures were performed by one trained operator.

For the study site, the teeth were cleaned and isolated using a rubber dam. A glass-ionomer-based sealant (RIVA Protect, SDI, Australia) was applied to the occlusal surface of the selected tooth after conditioning with 26% polyacrylic acid conditioner (RIVA-Conditioner, SDI, Australia) for 10 seconds, followed by abundant water washing and air drying. Excess water was removed, but the tooth was kept moist. The encapsulated material was prepared in an amalgamator (Silamat S5, Ivoclar Vivadent, Bendererstrasse) for 10 seconds and then applied with a dispensing gun. The material was gently extruded and spread onto the occlusal surface using a micro-brush. When the material had lost its surface gloss, a thin film of Riva-Coat was applied for 10 seconds. Light curing was carried out for 20 seconds using a DemiTM Plus light-curing unit (Kerr, Switzerland). Final finishing and occlusal adjustment, under water spray, was performed approximately after three minutes.

For the control site, the occlusal surface was etched with 35% phosphoric acid gel (Delton EZ etch, Dentsply, Germany) for 30 seconds. The etchant was gently stirred on the occlusal surfaces using a soft micro-brush, then rinsed for 30 seconds, and dried with an air syringe for five seconds. A resin-based sealant (Delton FS+, Dentsply, Germany) was applied directly onto the etched and dried surface with a round-ended applicator provided with the kit. In order to prevent overfilling, caution was exercised to avoid the contact of the applicator with the enamel surfaces. The sealant was left undisturbed for 20 seconds to allow its flow into the fissures and over the etched surface. Subsequently, the sealant was light-cured using a DemiTM Plus light-curing unit (Kerr, Switzerland) for 20 seconds. Occlusion was then checked with articulating paper, and adjustments were made using a finishing bur. The sealant was checked for complete coverage of all the pits and fissures and retention after complete polymerization with a fine probe. The parents and children were given age-appropriate dental health educational instructions, including proper brushing (twice a day, especially before going to bed), and proper flossing, if needed, was demonstrated on a model.

### Recall examination

Sealant retention and caries prevention were evaluated one week, three months, and six months after placement. Two calibrated investigators who were not involved in the treatment procedures visually evaluated the sealants using a mirror, a blunt explorer, and air syringe and reported scores for each tooth using the following criterion:

The effectiveness of sealants in preventing caries depended on long-term retention (Table 2).

**Table 2:** Criteria for clinical effectiveness of sealant (Simonsen, 1981; Bhushan and Goswami, 2017; Siripokkapat et al, 2018)

**Type**	**Criteria**
**Retention**	1. Complete retention of sealant: some peripheral fissures were uncovered following sealant wear, but no ledges were visible. 2. Partial loss of sealant: wear or material loss, part of a previously sealed pit/fissure was exposed. 3. Complete loss of sealant: no trace of sealant is detectable
**Caries transition**	Caries transition at the re-exposed surface where there is partial loss or total loss of sealant: 0. No change in caries: No change in the ICDAS score compared to the baseline and complete retention of sealant 1. Caries regression: A lower score than baseline 2. Caries progression: A higher score than baseline

### Statistical analysis

Data analysis was carried out using SPSS 21.0. The chi-squared test was used to determine whether there was a significant relationship between nominal (categorical) variables. Cramer's V was used to measure the strength of the association between the two nominal variables. A probability value of <0.05 at 95% CI was considered as statistically significant.

## Results

**Figure 1:**
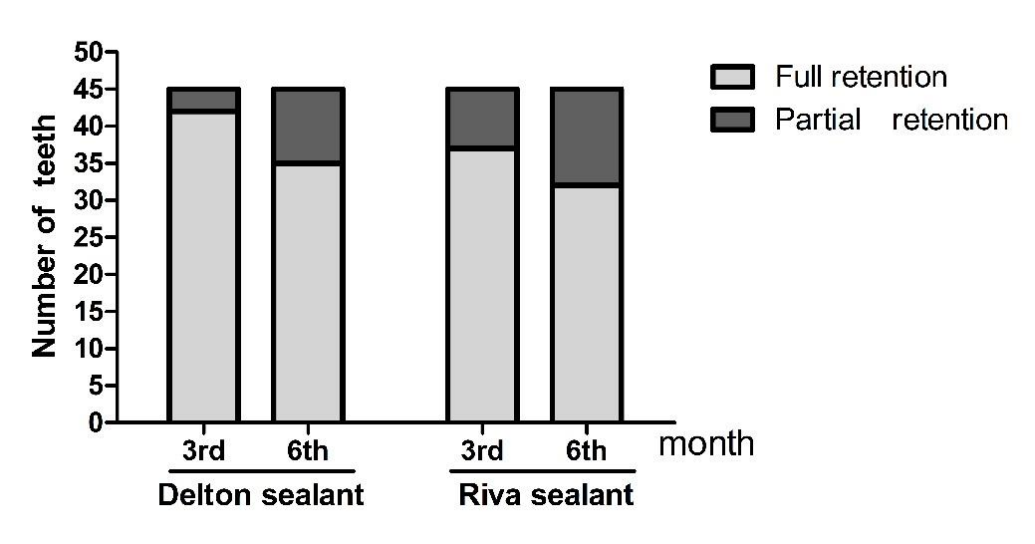
Comparison of retention of the sealant materials at three and six months.

Forty-five patients, 48% males and 52% females with a mean age of 10.09±1.411 years, participated in this study and were available at baseline as well as at all recall examination visits. The overall retention rate decreased significantly over three and six months in both sealant groups (P=0.0358 and P=0.0091 over three and six months, respectively). Table 3 presents sealants’ retention rates at three- and six-month recall examination visits. At three months, the RS group exhibited a significantly higher retention rate than the GIS group (93.33% and 77.77%, respectively). At six months, the RS group showed a higher percentage of retention rate than the GIS group (75.56% and 48.88%, respectively). The relationship between the type of sealant and retention rate was moderately significant (Cramer’s V= 0.22). At the six-month interval, the Delton group (75.56%) showed a higher percentage of full retention than the Riva-Protect group (48.88%). The difference in retention rates between the two groups, relative to baseline total number (P=0.0091), is presented in Table 3, Figure 1 (Table 4). The teeth treated with GIS had 3.23 times more significant risk to lose the sealant partially. Table 5 presents the details of caries transition over the study period.

**Table 3:** Comparison of the retention rate of the sealant materials at 3 and 6 months follow up

**Retention**	**3 months**		**6 months**	
	Delton sealant n(%)	Riva sealant n(%)	Delton sealant n(%)	Riva sealant n(%)
**Full retention**	42(93.33%)	35(77.77%)	34(75.56%)	22(48.88%)
**Partially loss**	3(6.67%)	10 (23.33%)	8(17.77%)	13(28.89%)
**Total**	45(100%)	45(100%)	42*(93.33%)	35^*^(77.77%)
**P value**	0.0358*		0.0091^*^	

* Original Data

**Table 4:** Sealant retention and caries transition at 3 and 6 months for both sealants’ types.

	**Delton sealant**				**Riva sealant**				**P-value**
	**Re-exposed teeth**				**Re-exposed teeth**				
**Month after sealant application**	**Complete** **sealant retention**	**No change**	**Caries** **regression**	**Caries** **prevention**	**Complete** **sealant retention**	**No change**	**Caries** **regression**	**Caries** **prevention**	
**3** ^rd^	42(93.33%)	2(4.44%)	0	44(97.7%)	35(77.78%)	8(17.78%)	1(2.22%)	44(97.7%)	1.00
**6** ^th^	34(75.56%)	8(17.78%)	0	42(93.33%)	22(48.88%)	8(17.78%)	0	30(66.65%)	0.0165*

* Original Data

**Table 5:** Caries status in the partially retained sealant by sealant type at 3^rd^ and 6^th^ month.

**Sealant type**	**Month**	**n**	**No change** **n( %)**	**Caries Progression** **n( %)**	**Caries regression** **n( %)**
**Delton**	3^rd^	3	2 (66.6%)	1(33.4%)	0
	6^th^	8	8(100%)	0	0
**Riva**	3^rd^	10	8 (80%)	1(10%)	1(10%)
	6^th^	13	8(61.5%)	5(38.5%)	0

*Original Data

## Discussion

The effectiveness of sealants depends on long-term retention and caries-preventive properties. In this clinical trial, we evaluated and compared the retention rate and caries transition of an RS and a GIS placed over non-cavitated carious occlusal pits and fissures of fully erupted permanent teeth using a split-mouth model. A split-mouth design was employed to reduce confounding factors, such as dietary behavior, caries risk, and oral hygiene practice in children.^10^ Many clinical trials have compared retention rates of different sealants and their effect on caries progression, most of which have compared different fissure sealants on partially erupted molars. When molars are fully erupted, the chance of sealant loss is higher due to the occlusal challenges. This might compromise sealant retention, restrict its ability to seal non-cavitated carious lesions, and prevent caries progression in high-risk patients. One of the limitations of investigating the effectiveness of sealants on partially erupted teeth is the difficulty of preventing moisture, which might lead to inconsistencies in the results. In this study, moisture control with a rubber dam was possible; thus, the effectiveness of the adhesive procedures and sealant placement would lead to more predictable results. Both materials were applied after enamel conditioning according to manufacturers’ instructions. In order to overcome the problem of early water uptake and improve the clinical performance of GIS, a surface coating agent has been used.^11,12^

The results of the retention rate revealed statistical significance between the two groups over six months of clinical evaluation. Therefore, the null hypothesis was rejected.

The resin-based sealant group exhibited a better retention rate than the GIS group (75.56% and 48.88%, respectively). The results of this study were contrary to earlier investigations.^13-15^ These investigators found no significant differences in retention rates between GIS and RS. Antonson et al^14^ attributed their results to the pretreatment of the fissures with a cavity conditioner. In our study, despite the moisture control and the surface pretreatment with cavity conditioner to improve adhesion, the low fluidity of GI sealant might have restricted its full penetration into the retentive fissures,^16^ making it more susceptible to partial or complete loss.

Additionally, it is well established that RS has superior physical properties as compared to GIS.^17^ The low abrasion resistance and the brittle nature of GIS, especially when placed on the occlusal surface of functioning teeth, might provide a reasonable explanation to our results. These findings were consistent with other studies performed on partially erupted teeth.^18-27^

Regarding caries development results, RS was superior to GIS in preventing caries progression, which might be attributed to the superior retention of the RS. Besides, the RS used in this study contains low-viscosity monomers and releasable sodium fluoride. This might help reduce acid attacks and bacterial levels while allowing the diffusion of calcium and phosphate ions to strengthen the tooth. The material composition, therefore, showed a double benefit regarding its high flow and fluoride release, which might explain its better performance when applied to fully erupted teeth.

Our results were supported by previous investigations by Radaal et al,^20^ Forss et al,^18^ and Poulsen et al,^28^ In contrast, Antonson et al,^14^ Haznedaroglu et al,^29^ and Haznedaroglu et al^30^ showed that GIS was better in preventing caries progression than RS. Antonson et al,^14^ who used the same RS (Delton FS) reported inferior results when compared to GIS. The discrepancy between the results is probably related to the method of application. They used the RS on the fissures of partially erupted molars under a moist condition. Apart from moisture, it is worth mentioning that in partially erupted molars the outer enamel layer is mostly prism-less, which might affect the etching efficacy and the marginal sealing ability.

Upon reviewing the literature on the effectiveness of fissure sealants in caries prevention, no evidence confirmed that one material is better than the other. The conflicting results of the laboratory, as well as clinical, studies have left the selection of the material up to the clinician’s preference. Although the results of laboratory studies can be good predictors of the clinical behavior of materials, long-term clinical studies are warranted to confirm our results. Additionally, diagnosing fissure caries, especially under a defective sealant, is often difficult without digital devices. These devices provide an effective quantitative analysis of caries progression and might be useful in research. Since caries is a chronic, slow, and insidious disease, long-term follow-up is more valuable to come up with a definite conclusion; the six-month follow-up can be considered as one of the limitations of this study.

## Conclusion

Within the limitations of this study, resin-based fissure sealant with fluoride releasing properties might be preferable than glass-ionomer-based sealant in preventing caries progression of incipient non-cavitated carious lesions in fully erupted teeth.

## Acknowledgments

None.

## Authors’ Contributions

JN: concept, design, definition of intellectual content, literature search, experimental studies, data acquisition, data analysis, statistical analysis, manuscript preparation, manuscript editing, and manuscript review. RH: supervision, design, definition of intellectual content, manuscript editing, and manuscript review. AA: supervision, design, definition of intellectual content, manuscript editing and manuscript review. EO: supervision, design, definition of intellectual content, manuscript editing, and manuscript review. All the authors have read and approved the final manuscript.

## Funding

None.

## Competing Interests

The authors declare no conflict(s) of interest related to the publication of this work.

## Ethics Approval

Approved by Beirut Arab University: 2018-H-0058-D-P-0258 on 31-july-2019.
